# Ionization States, Cellular Toxicity and Molecular Modeling Studies of Midazolam Complexed with Trimethyl-β-Cyclodextrin

**DOI:** 10.3390/molecules191016861

**Published:** 2014-10-21

**Authors:** Sergey Shityakov, Tamás Sohajda, István Puskás, Norbert Roewer, Carola Förster, Jens-Albert Broscheit

**Affiliations:** 1 Department of Anaesthesia and Critical Care, University of Würzburg, 97080 Würzburg, Germany; E-Mails: AN_Direktion@ukw.de (N.R.); Foerster_C@ukw.de (C.F.); Broscheit_J@ukw.de (J.-A.B.); 2 CycloLab Cyclodextrin Research & Development Laboratory Ltd., H-1097 Budapest, Hungary; E-Mails: sohajda@cyclolab.hu (T.S.); puskas@cyclolab.hu (I.P.)

**Keywords:** trimethyl-β-cyclodextrin, midazolam, transition state, molecular docking, Gibbs free energy of binding, quantum mechanics, free energy of solvation, torsional energy

## Abstract

We investigated the ionization profiles for open-ring (OR) and closed-ring (CR) forms of midazolam and drug-binding modes with heptakis-(2,3,6-tri-*O*-methyl)-β-cyclodextrin (trimethyl-β-cyclodextrin; TRIMEB) using molecular modeling techniques and quantum mechanics methods. The results indicated that the total net charges for different molecular forms of midazolam tend to be cationic for OR and neutral for CR at physiological pH levels. The thermodynamic calculations demonstrated that CR is less water-soluble than OR, mainly due to the maximal solvation energy (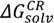 = −9.98 kcal·mol^−1^), which has a minimal 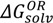 of −67.01 kcal·mol^−1^. A cell viability assay did not detect any signs of TRIMEB and OR/CR-TRIMEB complex toxicity on the cEND cells after 24 h of incubation in either Dulbecco’s Modified Eagles Medium or in heat-inactivated human serum. The molecular docking studies identified the more flexible OR form of midazolam as being a better binder to TRIMEB with the fluorophenyl ring introduced inside the amphiphilic cavity of the host molecule. The OR binding affinity was confirmed by a minimal Gibbs free energy of binding (*ΔG_bind_*) value of −5.57 ± 0.02 kcal·mol^−1^, an equilibrium binding constant (*K_b_*) of 79.89 ± 2.706 μM, and a ligand efficiency index (*LE_lig_*) of −0.21 ± 0.001. Our current data suggest that in order to improve the clinical applications of midazolam via its complexation with trimethyl-β-cyclodextrin to increase drug’s overall aqueous solubility, it is important to concern the different forms and ionization states of this anesthetic. All mean values are indicated with their standard deviations.

## 1. Introduction

Complexation mechanisms of drug-like chemical compounds with different cyclodextrins (CD) to establish a host-guest complex in solution, result in the improvement of pharmacokinetic parameters and physicochemical properties of the guest component, such as higher stability, increased aqueous solubility, decreased plasma protein binding, and cellular toxicity [1,2,3,4]. Molecular modelling and NMR studies have generated proposals on the mode of inclusion of the drug molecule by β-cyclodextrins [5,6] or their derivatives [7,8], including heptakis-(2,3,6-tri-*O*-methyl)-β-cyclodextrin or trimethyl-β-cyclodextrin, denoted as TRIMEB [9].

Midazolam is a preoperative anesthetic, belonging to a class of imidazobenzodiazepine compounds which is used as amnestics, hypnotics, anticonvulsants, and skeletal muscle relaxants [10] to further intensify the physiological repressive mechanisms mediated by γ-aminobutyric acid, the most common inhibitory neurotransmitter in the brain [11,12]. At acidic pH, midazolam exists over 90% in the open-ring (OR) and only 10% in the closed-ring (CR) form, reaching the equilibrium in the pH range 2.3–4.0 [13,14]. It is believed that the possible mechanism of the OR-to-CR conversion could be achieved via the ionized transition state (TS) intermediate by the simultaneous transfer of a hydrogen atom and the N=C bond formation [15].

At physiological pH, at least 99% of the mixture is presented mostly as CR form of this chemical compound, and it is also assumed that only this form of the benzodiazepines is pharmacologically active [16]. Usually, the OR form is produced in solution during the midazolam degradation revealing the dissimilarity in the photostability of this compound at different pH [14], which contributes to the overall aqueous solubility of the drug [17].

Previously, midazolam has been solubilized with various cyclodextrin derivatives, including hydroxypropyl-, sulfobutyl-, and randomly methylated modifications of β-cyclodextrin [15,18]. However, the overall complexation efficacy for cyclodextrins is frequently low in order to solubilize even small amounts of any given benzodiazepine drug [15].

Presently, there are no data theoretically or experimentally available on the interaction between different forms of midazolam and TRIMEB to assess the complexation rate and toxicity considering both drug ionization and solubilisation at different pH values. Therefore, the prime objective of the study was to investigate the ionization profiles and inclusion mechanisms of the different forms of midazolam bound to TRIMEB in conjunction with an assessment of the midazolam-TRIMEB cellular toxicity to diminish possible adverse effects.

## 2. Results and Discussion

**Figure 1:**
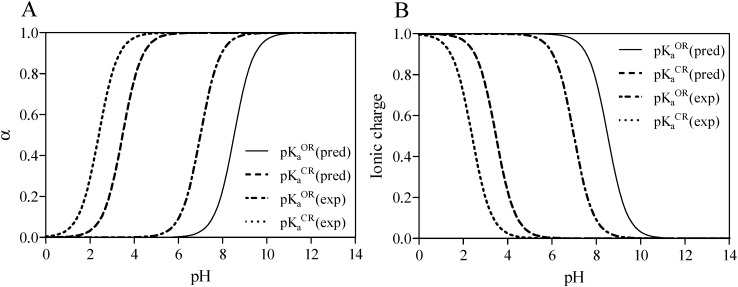
Calculated and experimentally derived degree of dissociation (**A**) denoted as α with ionic charge distribution (**B**) for the open-ring (OR) or closed-ring (CR) form of midazolam at different pH values according to Equations (1) and (2).

The aqueous solubility of midazolam plays an important role in the supramolecular complexation with water-soluble cyclodextrins and depends on the imidazobenzodiazepine ring opening, which is a fully reversible process, and the ionization of the drug molecule [15]. Therefore, the total charges were assessed from the acid dissociation constant determined for different forms of midazolam as depicted in Figure 1. Firstly, predicted negative decimal logarithm of acid dissociation constant for the nitrogen atom in position 1 (N-1) of OR (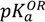 (pred)), CR (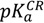 (pred)), and degree of dissociation for these forms of midazolam (Figure 1A) were calculated by using MarvinSketch software (ChemAxon, Budapest, Hungary). The net charges for midazolam forms at different pH were calculated from the Henderson-Hasselbalch equation [19] as follows:

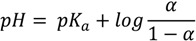
(1)
This equation could be rewritten to solve for α:

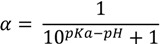
(2)
where *pK_a_* is the negative decimal logarithm of acid dissociation constant and *α* is the degree of dissociation (Supplementary material 1). The Equations (1) and (2) were implemented considering the charge-contributing functional groups for predicting molecular macrospecies distribution (88% for OR and 86% for CR) at physiological pH.

It was already experimentally determined for benzodiazepines that the positively charged N-1 atom (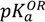 (exp) = 7.0, 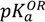 (pred) = 8.52) is affected by the pH-dependent reaction. Hence, the diazepine ring of 1,4-benzodiazepine moiety was formed with 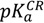 (exp) of 2.4 [15,20] and 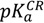 (pred) of 3.48. In addition, the basic nitrogen in position 2 of the imidazole ring, which belongs to the imidazobenzodiazepine moiety, allows the active elements of midazolam to form water-soluble salts with acids [21]. The imidazole ring of midazolam also accounts for its stability in solution and rapid metabolism [22]. Consequently, the calculated net charges for both molecular forms were found to be positive (+0.9) for the OR form of midazolam and benzodiazepines (+0.3) or neutral for the CR form at physiological pH (Figure 1B) and Supplementary material 2.

**Figure 2:**
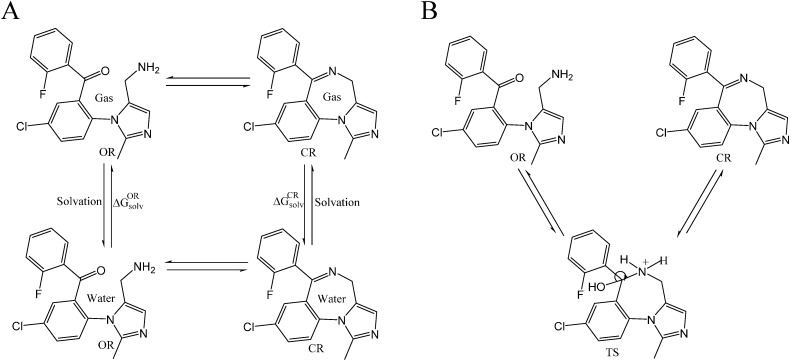
Thermodynamic cycle to evaluate the free energy of solvation (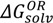 and 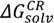) for midazolam open-ring (OR) and closed-ring (CR) forms (**A**) and reversible pH-dependent OR-to-CR conversion reaction (**B**) occurring through the formation of the ionized transition state intermediate (TS).

To further investigate the aqueous solubility, a restricted Hartree-Fock calculation with the 6-31G level of theory was performed using Pulay DIIS with geometric direct optimization to evaluate the free energy of solvation for the OR and CR forms of midazolam as shown in Figure 2A. In agreement with the experimental data that the aqueous solubility of midazolam at high pH defines by the formation of uncharged and lipophilic CR form [14], our calculations demonstrate that this structure has also been found to be less water-soluble because of higher lipophilicity (*ClogP* = 3.97), lower solvent excluded/accessible surface areas (*SES* = 278.56 Å^2^ and *SAS* = 518.59 Å^2^) and solvation energy (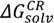 = −9.98 kcal·mol^−1^) compared to the OR form of midazolam with the *SES* (*SAS*) value of 296.68 (548.07) Å^2^, *ClogP* (ionic species) of 0.08, and 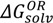 of −67.01 kcal·mol^−1^. For the OR/CR-TRIMEB inclusion complex, the optimal pH value to be easily dissolved is in the range from 3.5 to 3.7, which might imply some difficulties on its intravenous applications (unpublished data). Moreover, the further pH elevation in the solution (up to 7.0) might increase the risk of a suspension at physiological pH or even precipitate formation at basic pH value.

The complexation of midazolam in the OR, TS, and CR forms with TRIMEB for controlled drug delivery and sustained release have been also examined to assess its mechanism and estimate the effects involved in the simultaneous transfer of a hydrogen atom and C=N bond formation (Figure 2B). Given that methods like AutoDock have a typical error of ±2 kcal·mol^−1^, which might produce a huge deviation of *ΔG_bind_* and *K_b_* for the investigated compound [23], the midazolam-cyclodextrin complexes, including OR-TRIMEB, TS-TRIMEB, and CR-TRIMEB, were subjected to the molecular docking with the subsequent data collection for analysis from three different experiments (Supplementary material 3). The ionized TS intermediate used in the docking studies is only a rough representation of the “real” TS structure; therefore, the energy calculated for this species is quite approximate.

The molecular docking procedure as rigid-flexible approach using the Lamarckian genetic algorithm was already reported in various molecular docking studies as an efficient method to investigate ligand-cyclodextrin complexation mechanism [24,25,26]. On the other hand, the important guest-induced conformational change of the TRIMEB pyranose ring affecting the cavity shape and rim size [27,28] is not considered using our docking methodology due to the host total rigidity. Furthermore, the other obstacle to produce accurate free energy of complexation might be linked to the limit of the molecular mechanics AMBER force-field implemented in the AutoDock technique.

**Figure 3:**
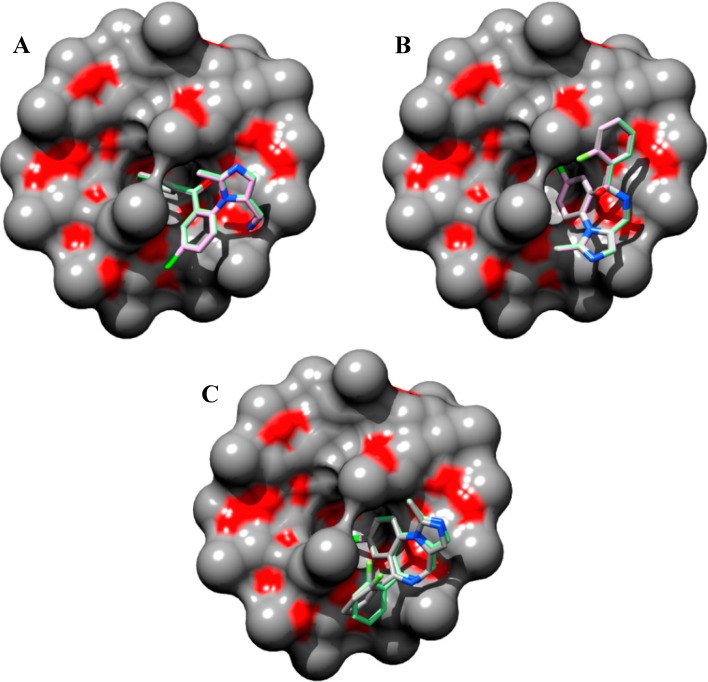
Predicted molecular docking modes with minimal Gibbs free energy of binding (*ΔG_bind_*) for open-ring (**A**), transition state intermediate (**B**) and closed-ring (**C**) form of midazolam complexed with TRIMEB. The molecular surface was reconstructed to visualize the binding cavity of uncharged trimethyl-β-cyclodextrin. The best binding poses with minimal *ΔG_bind_* values from three different molecular docking experiments are shown to reveal their conformational resemblances. Molecules are coloured according to their atom types. Hydrogen atoms are omitted for clarity.

Taken into account the modeled 1:1 stoichiometry of the open/closed-ring forms of midazolam and TRIMEB complex, it can be observed that the fluorine-containing phenyl ring of the OR structure, comprising big number of atoms (*N_ats_* = 26, *N_tor_* = 5), and chloride-containing diazepine ring in imidazobenzodiazepine moiety of TS (*N_ats_* = 27, *N_tor_* = 1) and CR (*N_ats_* = 23, *N_tor_* = 1) penetrate the TRIMEB cavity (Figure 3A–C).

In all cases, the degree of penetration for the guest molecule inside the host cavity is shallow. This might be caused by the partial occlusion of TRIMEB binding pocket with minimized states of methyl-glucose residues that under realistic conditions are expected to be quite mobile. In our docking experiments, the structure of TRIMEB represents “closed” spatial configuration when compared for instance with dimethyl-β-cyclodextrin or sulfobutyl-ether-β-cyclodextrin obtained by using several optimization steps in aqueous environment using the Merck molecular force-field (unpublished data).

Van Oudtshoorn and co-authors previously reported that the structure of the “free” TRIMEB molecule as minimized form adopted a severely collapsed conformation where the hydrophobic cavity is minimal in the absence of hydrophobic guest [29]. Furthermore, this distorted conformation might also be related to the conformation observed for the unfunctionalized β-cyclodextrin, which is stabilized by multiple intramolecular C-H···O interactions ubiquitously presented in crystal structure of carbohydrates [30,31]. In reality, the solvation effects on the extra methyl groups of the uncharged TRIMEB molecule might lead to the cavity re-opening process resulting in the larger volume size of the binding crevice.

**Figure 4:**
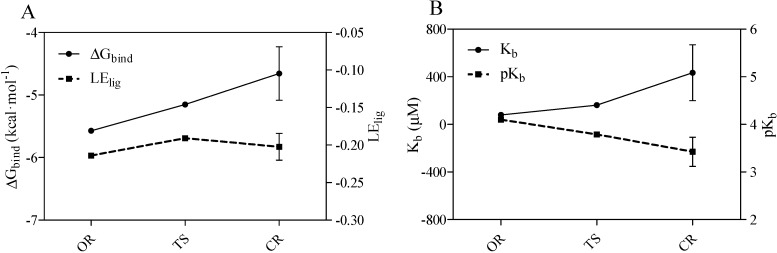
Predicted Gibbs free energy of binding (*ΔG_bind_*), ligand efficiency indexes (*LE_lig_*) (**A**) and equilibrium binding constants (*K_b_*/*pK_b_*) (**B**) for open-ring (OR) transition state intermediate (TS), and closed-ring (CR) ring forms of midazolam complexed with TRIMEB. The ligand efficiency is defined as the calculated *pK_b_* divided by the number of heavy atoms in the ligand. The data represent means ± S.D. of three independent experiments.

Finally, using the AutoDock program, the calculations for open-ring form of midazolam-TRIMEB complex provided the best binding affinity to TRIMEB detected for OR with an average *ΔG_bind_* value of −5.57 ± 0.02 kcal·mol^−1^ and an average *K_b_* constant of 79.89 ± 2.706 μM, respectively. Moreover, the pH-dependent OR-to-CR transition via the TS form followed the unflavored energetic path during the TRIMEB complexation and characterized by a gradual elevation in *ΔG_bind_* and *K_b_* (Figure 4A,B and Table 1). By increasing the number of returned docking poses (ga_run = 100), it was also determined that the originally provided docked pose is still the likely bound pose and there are 99 other possible host-guest configurations. Additionally, there are several commonly occurring docked poses formed during the clustering with the root-mean-square-deviation cut-off value of 2.0 Å and one of the most popular poses is the reported conformation with minimal *ΔG_bind_* value. In total, this low-energy conformation occurred in average 10, 36, and 34 times out of 100 for OR, TS, and CR forms, respectively (Supplementary material 3).

**Table 1:** Statistics of molecular docking results for open-ring (OR), transition state (TS), and closed-ring (CR) forms of midazolam formulated with TRIMEB.

	Average Value				SD			
Form	ΔG_bind_ *	LE_lig_	K_b_ (μM)	pK_b_	ΔG_bind_ *	LE_lig_	K_b_ (μM)	pK_b_
OR	−5.57	−0.21	79.89	4.09	0.02	0.001	2.706	0.015
TS	−5.15	−0.19	162.66	3.79	-	-	-	-
CR	−4.66	−0.2	434.05	3.43	0.427	0.018	235.034	0.308

Notes: *-Binding energies in kcal·mol^−1^; SD-Standard deviation.

**Figure 5:**
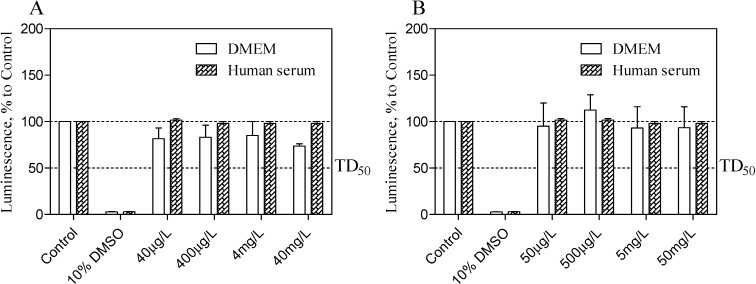
Cell viability assay is shown to measure the TRIMEB (**A**) and OR/CR-TRIMEB (**B**) toxic effects on the cEND cells over time (24 h). The luminescence is measured in percentage to a control group contained untreated cells. 10% solution of dimethyl sulfoxide (DMSO) was used as a positive control. The median toxic dose level is abbreviated as TD_50_. The thresholds are depicted as dashed lines. The data represent means ± S.D. of three independent experiments.

Taken into consideration the maximal clinical concentration for midazolam as 100 μg/L [32] and its relative inclusion content [w %], the CellTiter-Glo^®^ (Promega, Madison, WI, USA) luminescent cell viability assay was used to determine the cytotoxicity of TRIMEB and OR/CR-TRIMEB on the cEND cells in either DMEM or in heat-inactivated human serum. Although the high purification of TRIMEB was achieved with significantly improved aqueous solubility (>50 g in 100 cm^3^ at 25 °C), the powder might incorporate the traces of undermethylated-β-cyclodextrin and residual β-cyclodextrin (<0.25%). Cell viability was assessed by the amount of adenosine triphosphate (ATP) produced by metabolically active cells. The released ATP converts the luciferin substrate to luciferin oxide, and released luminescence signals were recorded. The results of this assay showed the absence of a toxic effect of analysed substances in either DMEM or in human serum on cEND cells after 24 h of incubation. Overall, with increased time, no significant difference from the actual cell viability for TRIMEB and OR/CR-TRIMEB was revealed; their luminescence levels remained above the median toxic dose level (TD_50_). A significant reduction in luminescence activity was discovered after the treatment with 10% dimethyl sulfoxide (DMSO) served as a positive control indicating a massive cell death (Figure 5A,B).

**Figure 6:**
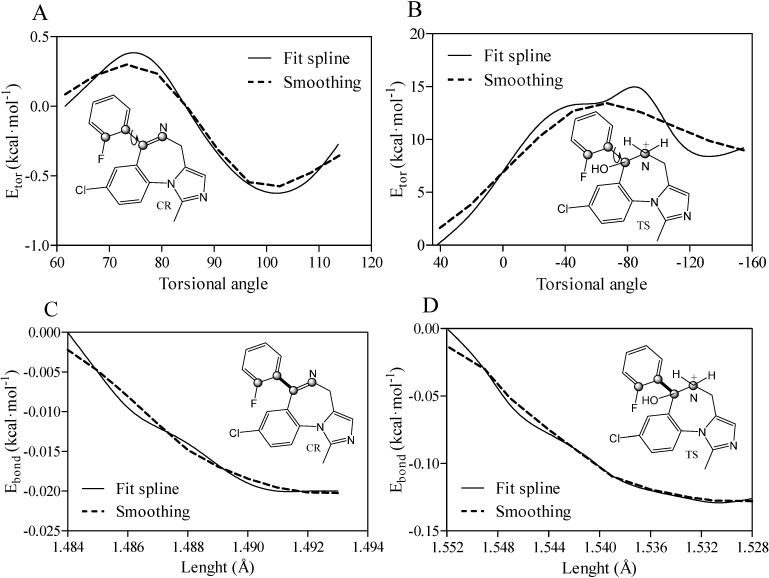
Predicted relative torsional (*E_tor_*) energy (**A**,**B**) and bond length (*E_bond_*) energy (**C**,**D**) for closed-ring (OR) and transition state (TS) forms of midazolam complexed with TRIMEB. A fit spline and smoothing curves are implemented to connect and fit the data points. All atoms comprising the torsional angle are depicted as grey spheres and measured bonds are illustrated as bold lines.

To gain some insight into the binding characteristics of midazolam as unionized closed-ring molecule with a single torsional angle (N1-C7-C1-C2 framework), the quantum chemical method was implemented to evaluate the torsional energy contribution to the complexation process as a repulsion force between the bonds of groups attached to a central rotating bond. In particular, the Hartree-Fock technique with the 6-31G level of theory was applied to set the dihedral N1-C7-C1-C2 angle from 61.6° determined for the minimized ligand to 114.23° increments determined for the best docking pose. The angular rotation occurred in a clockwise direction to record the energy for each point in a vacuum environment. The relative *E_tor_* values for all the different conformations of CR (10 structures) were in the range from −0.624 to 0.377 kcal·mol^−1^ (rotation is free at room temperature) indicating energetically favored and unflavored planar positions of the fluorophenyl ring and imidazobenzodiazepine moiety at different angles. Finally, the *E_tor_* value of −0.25 kcal·mol^−1^ was estimated for the best docking pose with the minimal *ΔG_bind_* parameter resulting in the molecular interface rotation to reach the lowest energy orientation in comparison to the minimized structure (Figure 6A). Additionally, the relative energy contribution for the positively charged TS intermediate in the course of the complexation was also evaluated as a function of the internal rotation around the same dihedral angle. The TS torsional angle shifts from 42.42° to −154.49° with the total increase in the relative torsional energy (*E_tor_* = 9.21 kcal·mol^−1^). This increase in the *E_tor_* value might be due to the steric hindrance between the hydroxyl side chain moiety and the fluorophenyl ring (Figure 6B). While a negative shift of −196.91° requiring an anticlockwise rotation prevails in the transition state as antiperiplanar or gauche-conformation, the increase in total angular shift between the same molecular parts was about +52.63° for OR corresponding to a positive anticlinal arrangement. Moreover, the intramolecular C7-C1 bond of CR was extended by 0.011 Å for the constrained bond length in the range from 1.484 to 1.495 Å, and a decrease in the bond length energy (*E_bond_* = −0.08 kcal·mol^−1^) was also observed (Figure 6C). Conversely, the C7-C1 bond distance in the TS structure was diminished by 0.024 Å from 1.552 to 1.528 Å with a negative bond length energy of −0.53 kcal·mol^−1^ (Figure 6D). Consequently, the TS bond compression facilitated the simultaneous transfer of a hydrogen atom and N1C7 double bond formation along with its influence on the fluorophenyl ring rotation.

## 3. Experimental Section

The pure forms of TRIMEB (98% of purity) and midazolam-TRIMEB complex (OR/CR-TRIMEB) with 18.2% [w %] midazolam relative content to OR (22%) and CR (78%) fractions were prepared according to the following procedure: anhydrous β-CD (6.0 g, Sigma-Aldrich Co., St. Louis, MO, USA) was dissolved in dry dimethylformamide (50 mL). Sodium hydride (8.2 g) was added, and the mixture was stirred at room temperature for 20 min. The jelly-like mixture was cooled to 0 °C and treated portionwise with methyl iodide (43.6 mL) over a period of 30 min; the insoluble materials went into solution. The solution was stirred at room temperature for 24 h, cooled and the excess of sodium hydride was decomposed by the addition of methanol (25 mL). Finally, a white, amorphous solid powder of TRIMEB was obtained by hydrolysation and acetylation under conventional conditions for 6.45 min at room temperature [33].

TRIMEB (10.77 g, 7.5 mM) was dissolved in 80 mL of distilled water. Midazolam (2.45 g, 7.5 mM) was added to the TRIMEB solution. The resulting suspension was stirred, and the pH value of the mixture was adjusted to 3.2 by adding 0.5 M hydrochloric acid (10.5 mL). The clear solution was stirred for 10 min and filtered through a hydrophilic polyvinylidene fluoride filter with 0.45 μm pore size. Subsequently, the final solution was frozen and lyophilized.

**Figure 7:**
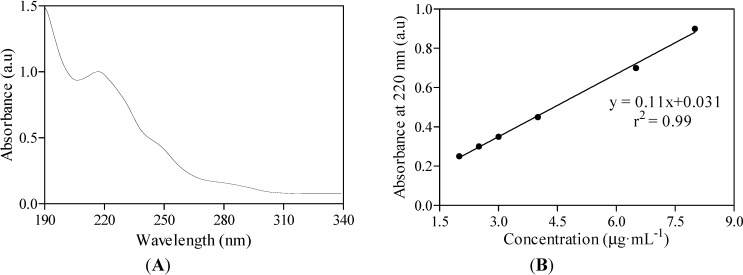
UV-Vis spectrum of midazolam as OR/CR mixture based on the absorbance peak found at wavelength 220 nm (**A**). Midazolam aqueous solution is examined in 50% of ethanol at a pH level of 3.0. The absorbance is measured in arbitrary units, which are abbreviated as a.u. The linear calibration curve was fitted to the experimental absorbance data (**B**).

An Agilent 8453 Diode Array Spectrophotometer was used for UV-Vis spectroscopic quantification of the drug substance within the complex in a quartz cuvette of 1 cm path length (Figure 7A,B).

**Figure 8:**
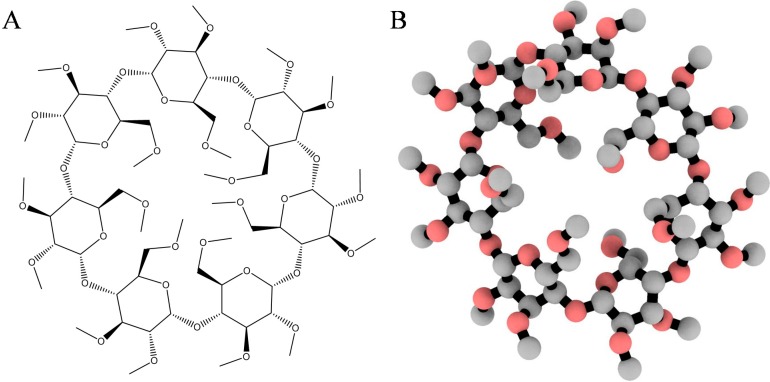
Two- (**A**) and three-dimensional (**B**) representation of the heptakis-(2,3,6-tri-*O*-methyl)-β-cyclodextrin (TRIMEB) structure. The 3D model is rendered as ball-and-stick and coloured according to atom type without hydrogens to enhance clarity.

The two-dimensional coordinates of heptakis-(2,3,6-tri-*O*-methyl)-β-cyclodextrin (Figure 8A) or TRIMEB (CAS No. 55216-11-0) and the closed-ring form of midazolam (CID: 4192) were retrieved from the Chemical Book and PubChem servers, converted into the energetically minimized three-dimensional models (Figure 8B) using MarvinSketch software (ChemAxon, Budapest, Hungary).

The same program was implemented to build the OR form and ionized TS intermediate, to perform their energy minimization and predict the calculated octanol-water partitioning coefficient (*ClogP*) with a weighted method as the default option and ion (Cl^−^, Na^+^, and K^+^) concentration of 0.1 mol·dm^−3^. Rigid-flexible molecular docking was applied to the centre of the cyclodextrin molecule using Cartesian coordinates: x = −0.78 Å; y = −0.17 Å; and z = 0.0 Å. AutoDock v.4.2.5.1 was used in the study since its previous version incorrectly calculates part of the intermolecular desolvation energy term. The docking grid with a dimension size of 30 Å × 30 Å × 30 Å was used in the study. The grid spacing of 0.375 Å was used to create the AutoDock grid maps. For each compound, a number of standard genetic algorithm dockings (ga_run) was set to 100. The authors used the default settings within AutoDock and AutoGrid. The host and guest structure preparations for molecular docking included Gasteiger partial charges assignment [34] and rotatable bonds definition. The docking output results were represented by the docking scores as the estimated Gibbs free energy of binding (*ΔG_bind_*) and were further converted to the predicted equilibrium binding constants (*K_b_*) or *pK_b_* values (negative decimal logarithm of *K_b_*). Estimated *ΔG_bind_* and *K_b_* parameters for the docked poses were calculated using the equations shown below:

*ΔG_bind_* = *E_inter_* + *E_internal_* + *E_tor_* − *E_unbound_* (3)

*E_inter_* = *E_vdw_* + *E_hb_* + *E_desolv_* + *E_elect_* (4)

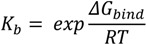
(5)
where *E_inter_* and *E_internal_* are the final intermolecular and total internal energies; *E_vdw_hd_desolv_* corresponds to the sum of the energies of van der Waals (vdw), hydrogen bond (hb), and desolvation (desolv) terms; *E_elect_* is the electrostatic energy; *E_tor_* and *E_unbound_* are the torsional free and unbound system’s energies; R (gas constant) is 1.98 cal(mol·K)^−1^, and T (room temperature) is 298.15 Kelvin, respectively. The solvent excluded surface (*SES*) area and solvent accessible surface (*SAS*) area were calculated by the MGLTools program (Scripps Research Institute, San Diego, CA, USA); the python script (summarize_results4.py) was used to analyze and summarize the AutoDock results. A ligand efficiency index (*LE_lig_*) was computed as a parameter recently introduced for selection of useful lead molecules according to their binding energy per atom [35,36] using the following equation:

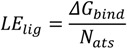
(6)
where *ΔG_bind_* is the Gibbs free energy of binding and *N_ats_* is the number of heavy atoms.

To determine the cell viability for the pure TRIMEB structure and OR/CR-TRIMEB complex, the immortalized microvascular endothelial (cEND) cells of murine origin [37,38,39] were seeded in 96-well plate and grown to 90% of confluency in Dulbecco’s Modified Eagles Medium (DMEM) containing 10% fetal calf serum (FCS), 50 U/mL penicillin/streptomycin, and 1% L-glutamine. Once confluent, cells were forced to differentiate in 1% serum-stripped fetal calf serum (ssFCS) for two days. Next, cells were incubated with analyzed substances in final concentrations of 40 µg/L–40 mg/L (TRIMEB) and 50 µg/L–50 mg/L (OR/CR-TRIMEB) for 24 h at 37 °C. Afterwards, cell viability was assessed using the CellTiter-Glo^®^ (Promega, Madison, WI, USA) luminescent cell viability assay kit according to the manufacturer’s recommendation. Briefly, the test compound and controls were added to the cells in either DMEM or in heat-inactivated (56 °C for 30 min) human serum and the cells were further incubated for 30 min at room temperature, which after the CellTiter-Glo^®^ solution was added. Cell lysis was achieved for 2 min with shaking followed by 10 min of equilibration at room temperature. Luminescence was assayed using the Tecan GENios Microplate Reader (MTX Lab Systems, Inc., Vienna, VA, USA). The restricted Hartree-Fock single-point energy calculations were performed with Pulay DIIS model and geometric direct optimization as implemented in the Spartan software (Wavefunction, Inc., Irvine, CA, USA) in order to calculate the free energy of solvation for OR/CR forms of midazolam (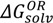, 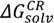), relative torsional (*E_tor_*), and bond length (*E_bond_*) energies. Relative torsional energy *E_tor_* was calculated from a truncated Fourier series using the equation in the form shown below:

*E_tor_* = *k_tor_*_1_ (1 − *cos*(*ω* − *ω_eq_*)) + *k_tor_*_2_ (1 − *cos*2(*ω* − *ω_eq_*)) + *k_tor_*_3_ (1 − *cos*3(*ω* − *ω_eq_*)) (7)

where *ω_eq_* is the ideal dihedral angle and *k*_*tor*1_, *k*_*tor*2_, and *k*_*tor*3_ are the torsional constants for one-fold, two-fold, and three-fold rotational barrier. Fourier series were implemented to solve nonlinear least squares curve-fitting via the oscillating function. Bond length energy (stretching and compression) based on Hook’s law was computed as follows:

*E_bond_* = Σ *bondsk_b_* (*r* − *r*_0_)^2^ (8)

where *k_b_* is the stiffness factor while *r* and *r*_0_ values define the bond equilibrium length for each bonded atoms based on their types. All molecular rendering scenes and graphic representations were prepared by the Persistence of Vision Raytracer (POV-Ray) module (Persistence of Vision Pty. Ltd., Williamstown, Australia) included in the Chimera version 1.6.2 software (Resource for Biocomputing, Visualization, and Informatics, San Francisco, CA, USA), ChemBioDraw^®^ Ultra 14 (PekinElmer, Waltham, MA, USA), QuteMol v.0.4.1 [40], Wolfram Mathematica 10 (The Wolfram Centre, Long Hanborough, UK), and GraphPad Prism v.4 for Windows software (GraphPad Software, Inc., San Diego, CA, USA).

## 4. Conclusions

In the current study, we report results that show that the total net charges have a tendency to be cationic for OR and neutral for CR at physiological pH, influencing their complexation with the TRIMEB molecule. A lower degree of aqueous solubility was found for the CR structure with its higher solvation energy (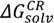
= −9.98 kcal·mol^−1^) than for the more hydrophilic OR form with a minimal 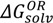 of −67.01 kcal·mol^−1^ during the OR-to-CR conversion that occurred through the formation of ionized TS intermediate. The absence of TRIMEB and OR/CR-TRIMEB toxicity in the cEND cells after 24 h of incubation (in either Dulbecco’s Modified Eagles Medium or heat-inactivated human serum) was confirmed by a CellTiter-Glo^®^ (Promega) luminescent cell viability assay. Despite the lack of cellular toxicity, the native pH value required to dissolve the OR/CR-TRIMEB complex is in the range of 3.5–3.7, limiting its usage to clinical applications. The molecular docking method detected that the more flexible OR form (*N_tor_* = 5) of midazolam may serve as a better binder to trimethyl-β-cyclodextrin with the fluorophenyl ring introduced inside the amphiphilic cavity of the TRIMEB. The optimal OR binding affinity was verified by a minimal *ΔG_bind_* value of −5.57 ± 0.02 kcal·mol^−1^, an equilibrium binding constant (*K_b_*) of 79.89 ± 2.706 μM, and a ligand efficiency index (*LE_lig_*) of −0.21 ± 0.001. A decrease in the torsional energy (*E_tor_* = −0.25 kcal·mol^−1^) for the active CR form was detected in order to reach the lowest energy orientation during the conformational sampling. Therefore, it is important to improve the clinical applications of midazolam via its complexation with trimethyl-β-cyclodextrin in order to increase its overall aqueous solubility concerning the different forms and ionization states of this anaesthetic.

## Supplementary Materials

Supplementary materials can be accessed at: http://www.mdpi.com/1420-3049/19/10/16861/s1.

## Supplementary Files

Supplementary File 1
