# Supplementary material for: Ionization States, Cellular Toxicity and Molecular Modeling Studies of Midazolam Complexed with Trimethyl-β-Cyclodextrin
Source: Molecules. 2014 Oct 21;19(10):16861–76. doi: 10.3390/molecules191016861 (PMC6270744; doi:10.3390/molecules191016861)

Supporting information

Supplementary Material 1: Figures S1,S2

Figure S1. Predicted acid dissociation constants (pKa) for the open-ring (OR) and closed-ring (CR) forms of midazolam. The pKa values are assigned to the nitrogen atoms at physiological pH. At pH of 7.36, the diazepine ring closes and the chemical substance becomes highly lipophilic.

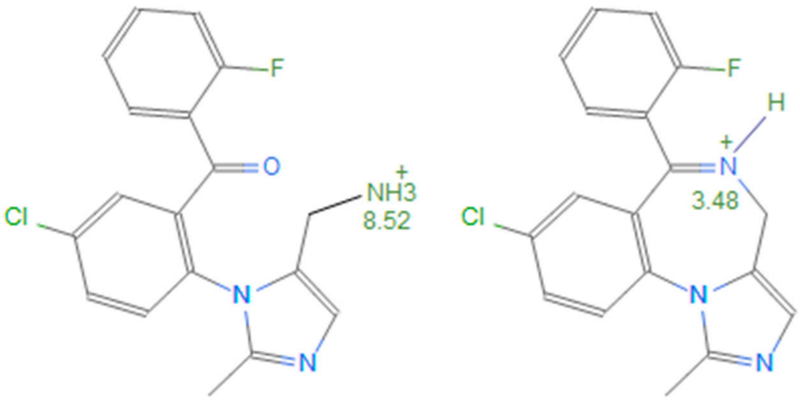

OR CR

Figure S2. Mathematica script to determine degree of dissociation (α) for open-ring (OR) and closed-ring (CR) forms of midazolam and benzodiazepines according to Henderson-Hasselbalch equation.

```
y = ((1 + 10^(8.52 - x)))^(-1)
```

$$\frac{1}{1 + 10^{8.52 - x}}$$

Show[%53, AxesLabel → {None, Nine}, FrameLabel → {{HoldForm[α], None}, {HoldForm[pH], None}}, PlotLabel → None, LabelStyle → {GrayLevel[0]}]

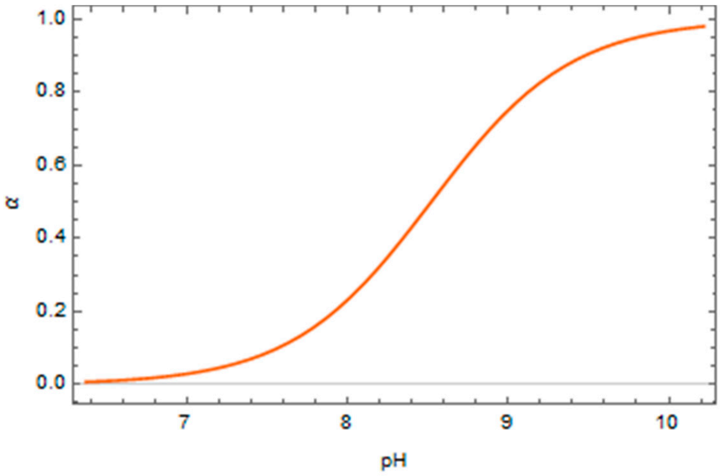


```
y = ((1 + 10^(3.48 - x)))^(-1)
```

$$\frac{1}{1 + 10^{3.48 - x}}$$

Show[%60, AxesLabel → {None, Nine}, FrameLabel → {{HoldForm[α], None}, {HoldForm[pH], None}}, PlotLabel → None, LabelStyle → {GrayLevel[0]}]

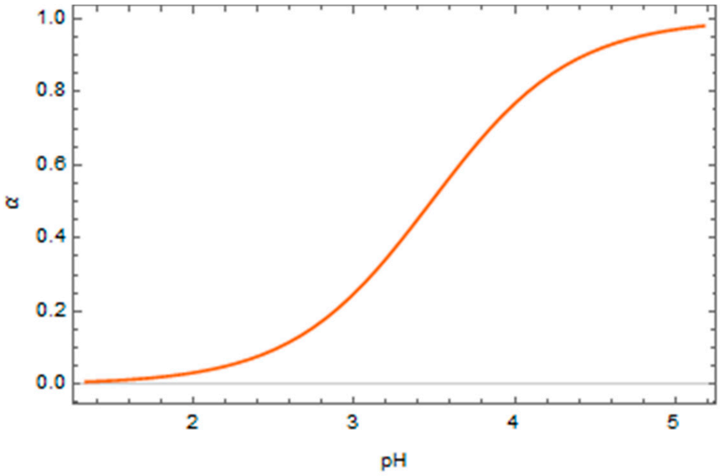


```
y = ((1 + 10^(7.0 - x)))^(-1)
```

$$\frac{1}{1 + 10^{7. - x}}$$

Show[%66, AxesLabel → {None, Nine}, FrameLabel → {{HoldForm[α], None}, {HoldForm[pH], None}}, PlotLabel → None, LabelStyle → {GrayLevel[0]}]

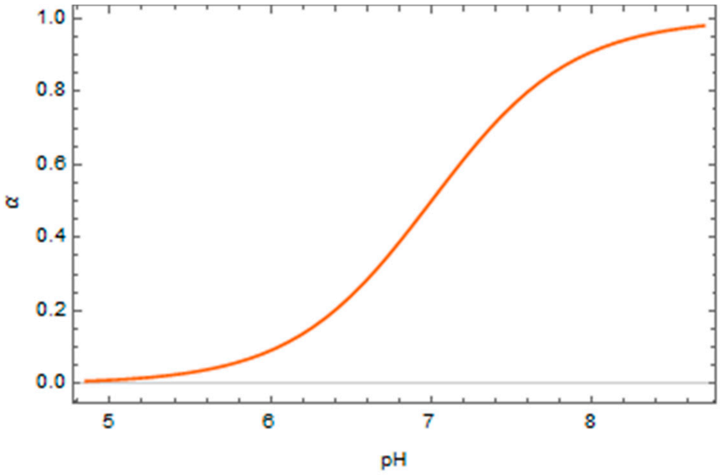


```
y = ((1 + 10 ^ (2.4 - x))) ^ (-1)
```

$$\frac{1}{1 + 10^{2.4-x}}$$

Show[%71, AxesLabel → {None, Nine}, FrameLabel → {{HoldForm[α], None}, {HoldForm[pH], None}}, PlotLabel → None, LabelStyle → {GrayLevel[0]}]

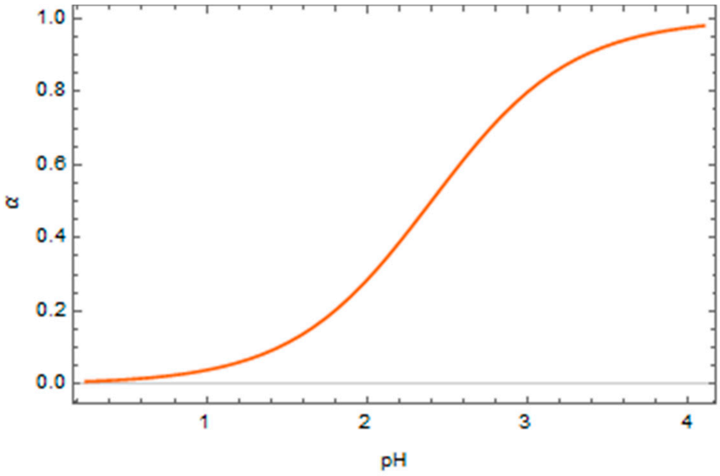

Supplement: Supplementary File 1 [file molecules-19-16861-s001.zip › Supplementary Material 1.pdf]
