# Supplementary material for: Ionization States, Cellular Toxicity and Molecular Modeling Studies of Midazolam Complexed with Trimethyl-β-Cyclodextrin
Source: Molecules. 2014 Oct 21;19(10):16861–76. doi: 10.3390/molecules191016861 (PMC6270744; doi:10.3390/molecules191016861)
Supplement: Supplementary File 1 [file molecules-19-16861-s001.zip › Supplementary Material 2.pdf]

Supporting information

Supplementary Material 2: Figures S3,S4

Figure S3. Mathematica script to determine net charge distribution for open-ring (OR) and closed-ring (CR) forms of midazolam and benzodiazepines according to Henderson-Hasselbalch equation.

```
y = ((-1./((10^(8.52 - x)) + 1)) + 1)
```

$$1 - \frac{1.}{1 + 10^{8.52-x}}$$

Show[%3, AxesLabel → {None, Nine}, FrameLabel → {{HoldForm[Charge], None}, {HoldForm[pH], None}}, PlotLabel → None, LabelStyle → {GrayLevel[0]}]

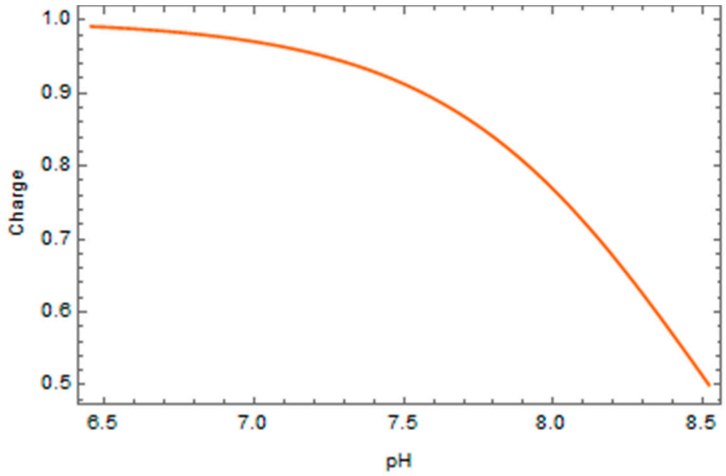


```
y = ((-1./((10^(3.48 - x)) + 1)) + 1)
```

$$1 - \frac{1.}{1 + 10^{3.48-x}}$$

Show[%13, AxesLabel → {None, Nine}, FrameLabel → {{HoldForm[Charge], None}, {HoldForm[pH], None}}, PlotLabel → None, LabelStyle → {GrayLevel[0]}]

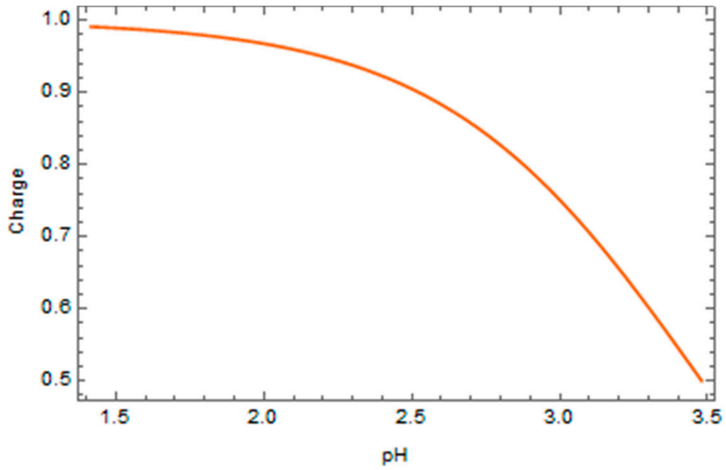


```
y = ((-1./((10^(7.0 - x)) + 1)) + 1)
```

$$1 - \frac{1.}{1 + 10^{7.-x}}$$

Show[%18, AxesLabel → {None, Nine}, FrameLabel → {{HoldForm[Charge], None}, {HoldForm[pH], None}}, PlotLabel → None, LabelStyle → {GrayLevel[0]}]

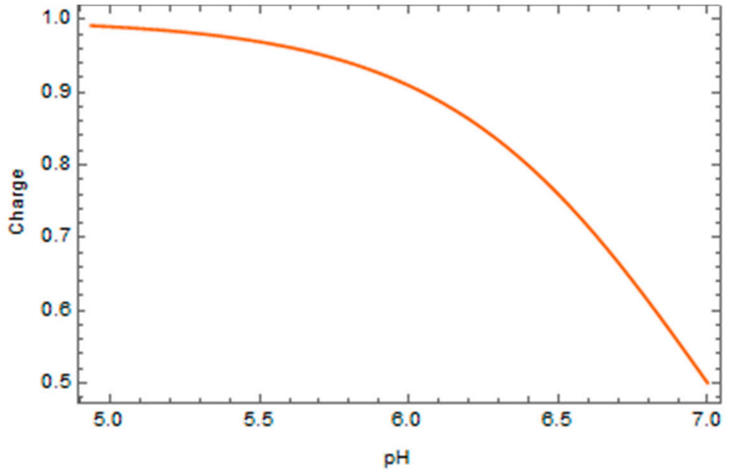


```
y = ((-1. / ((10^(2.4 - x)) + 1)) + 1)
```

$$1 - \frac{1.}{1 + 10^{2.4-x}}$$

Show[%25, AxesLabel → {None, Nine}, FrameLabel → {{HoldForm[Charge], None}, {HoldForm[pH], None}}, PlotLabel → None, LabelStyle → {GrayLevel[0]}]

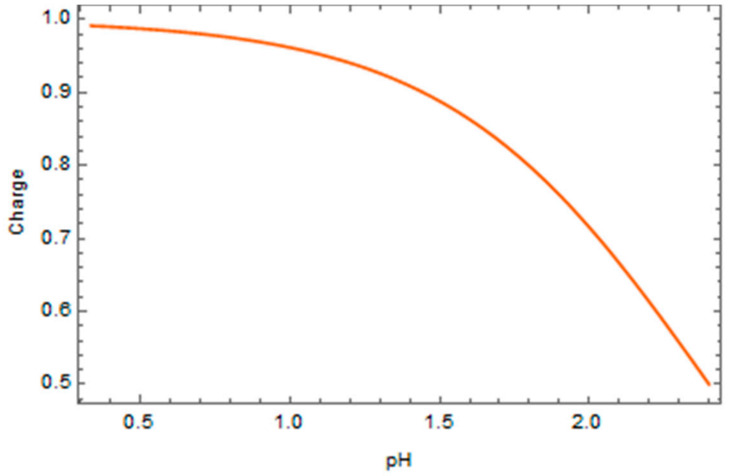

Figure S4. Mathematica script to determine net charge for open-ring (OR) and closed-ring (CR) forms of midazolam and benzodiazepines according to Henderson-Hasselbalch equation at physiological pH.

```
y = ((-1. / ((10^(8.52 - 7.36)) + 1)) + 1)
0.935293
y = ((-1. / ((10^(3.48 - 7.36)) + 1)) + 1)
0.000131808
y = ((-1. / ((10^(7.0 - 7.36)) + 1)) + 1)
0.303871
y = ((-1. / ((10^(2.4 - 7.36)) + 1)) + 1)
0.0000109647
```
