# Supplementary material for: Ionization States, Cellular Toxicity and Molecular Modeling Studies of Midazolam Complexed with Trimethyl-β-Cyclodextrin
Source: Molecules. 2014 Oct 21;19(10):16861–76. doi: 10.3390/molecules191016861 (PMC6270744; doi:10.3390/molecules191016861)

**Supporting information**

**Supplementary Material 3:** Tables S1–S3, Figures S5–S7

**Table S1.** Molecular docking results from three different experiments for open-ring (OR) form of midazolam formulated with TRIMEB.

| Rank | Form | Pose | $\Delta G_{bind}$ | Pose | $\Delta G_{bind}$ | Pose | $\Delta G_{bind}$ |
|---|---|---|---|---|---|---|---|
| 1 | OR | 1 | -5.590 | 1 | -5.550 | 1 | -5.570 |
| 2 | OR | 2 | -5.540 | 2 | -5.140 | 2 | -5.110 |
| 3 | OR | 3 | -5.360 | 3 | -4.990 | 3 | -4.960 |
| 4 | OR | 4 | -5.000 | 4 | -4.950 | 4 | -4.710 |
| 5 | OR | 5 | -4.940 | 5 | -4.870 | 5 | -4.600 |
| 6 | OR | 6 | -4.910 | 6 | -4.780 | 6 | -4.510 |
| 7 | OR | 7 | -4.810 | 7 | -4.730 | 7 | -4.390 |
| 8 | OR | 8 | -4.800 | 8 | -4.620 | 8 | -4.320 |
| 9 | OR | 9 | -4.590 | 9 | -4.560 | 9 | -4.310 |
| 10 | OR | 10 | -4.590 | 10 | -4.460 | 10 | -4.290 |
| 11 | OR | 11 | -4.510 | 11 | -4.340 | 11 | -4.250 |
| 12 | OR | 12 | -4.470 | 12 | -4.260 | 12 | -4.240 |
| 13 | OR | 13 | -4.470 | 13 | -4.240 | 13 | -4.220 |
| 14 | OR | 14 | -4.380 | 14 | -4.230 | 14 | -4.210 |
| 15 | OR | 15 | -4.310 | 15 | -4.180 | 15 | -4.160 |
| 16 | OR | 16 | -4.270 | 16 | -4.170 | 16 | -4.060 |
| 17 | OR | 18 | -4.250 | 17 | -4.160 | 17 | -4.010 |
| 18 | OR | 17 | -4.250 | 18 | -4.120 | 18 | -3.910 |
| 19 | OR | 19 | -4.240 | 19 | -3.940 | 19 | -3.890 |
| 20 | OR | 20 | -4.210 | 20 | -3.920 | 20 | -3.880 |
| 21 | OR | 21 | -4.180 | 22 | -3.880 | 21 | -3.820 |
| 22 | OR | 23 | -4.140 | 21 | -3.880 | 22 | -3.810 |
| 23 | OR | 22 | -4.140 | 23 | -3.830 | 23 | -3.770 |
| 24 | OR | 24 | -4.140 | 24 | -3.620 | 24 | -3.760 |
| 25 | OR | 25 | -4.130 | 25 | -3.560 | 25 | -3.700 |
| 26 | OR | 26 | -4.070 | 26 | -3.530 | 26 | -3.690 |
| 27 | OR | 27 | -4.040 | 27 | -3.420 | 27 | -3.680 |
| 28 | OR | 28 | -3.900 | 28 | -3.340 | 28 | -3.630 |
| 29 | OR | 29 | -3.880 | 29 | -3.320 | 29 | -3.620 |
| 30 | OR | 30 | -3.820 | 30 | -3.310 | 30 | -3.590 |
| 31 | OR | 31 | -3.790 | 31 | -3.290 | 31 | -3.550 |
| 32 | OR | 32 | -3.710 | 32 | -3.290 | 32 | -3.550 |
| 33 | OR | 33 | -3.680 | 33 | -3.250 | 33 | -3.540 |
| 34 | OR | 34 | -3.610 | 34 | -3.240 | 34 | -3.480 |
| 35 | OR | 35 | -3.570 | 35 | -3.240 | 35 | -3.470 |
| 36 | OR | 36 | -3.540 | 36 | -3.240 | 36 | -3.450 |
| 37 | OR | 37 | -3.450 | 37 | -3.200 | 37 | -3.440 |
| 38 | OR | 38 | -3.380 | 38 | -3.180 | 38 | -3.440 |
| 39 | OR | 39 | -3.380 | 39 | -3.140 | 39 | -3.430 |
| 40 | OR | 40 | -3.360 | 40 | -3.130 | 40 | -3.380 |
| 41 | OR | 41 | -3.250 | 41 | -3.120 | 41 | -3.330 |
| 42 | OR | 43 | -3.250 | 42 | -3.080 | 42 | -3.300 |

**Table S1.** *Cont.*

| Rank | Form | Pose | ΔG$_{bind}$ | Pose | ΔG$_{bind}$ | Pose | ΔG$_{bind}$ |
|---|---|---|---|---|---|---|---|
| 43 | OR | 42 | -3.250 | 44 | -3.070 | 43 | -3.270 |
| 44 | OR | 44 | -3.240 | 43 | -3.070 | 44 | -3.260 |
| 45 | OR | 45 | -3.200 | 45 | -3.060 | 45 | -3.250 |
| 46 | OR | 46 | -3.150 | 46 | -3.040 | 46 | -3.240 |
| 47 | OR | 47 | -3.130 | 47 | -3.020 | 47 | -3.110 |
| 48 | OR | 48 | -3.110 | 49 | -2.950 | 48 | -3.110 |
| 49 | OR | 49 | -3.090 | 48 | -2.950 | 49 | -3.100 |
| 50 | OR | 50 | -3.050 | 50 | -2.770 | 50 | -3.080 |
| 51 | OR | 51 | -3.030 | 51 | -2.690 | 51 | -3.080 |
| 52 | OR | 52 | -3.010 | 52 | -2.680 | 52 | -3.070 |
| 53 | OR | 53 | -2.990 | 53 | -2.630 | 53 | -3.020 |
| 54 | OR | 54 | -2.960 | 54 | -2.620 | 56 | -2.990 |
| 55 | OR | 55 | -2.960 | 56 | -2.560 | 54 | -2.990 |
| 56 | OR | 56 | -2.950 | 55 | -2.560 | 55 | -2.990 |
| 57 | OR | 57 | -2.860 | 57 | -2.550 | 58 | -2.960 |
| 58 | OR | 58 | -2.830 | 58 | -2.520 | 57 | -2.960 |
| 59 | OR | 59 | -2.820 | 61 | -2.510 | 60 | -2.910 |
| 60 | OR | 60 | -2.800 | 60 | -2.510 | 59 | -2.910 |
| 61 | OR | 61 | -2.720 | 59 | -2.510 | 61 | -2.890 |
| 62 | OR | 62 | -2.710 | 62 | -2.480 | 62 | -2.850 |
| 63 | OR | 63 | -2.630 | 64 | -2.380 | 63 | -2.820 |
| 64 | OR | 66 | -2.590 | 63 | -2.380 | 64 | -2.780 |
| 65 | OR | 65 | -2.590 | 65 | -2.330 | 65 | -2.680 |
| 66 | OR | 64 | -2.590 | 66 | -2.320 | 66 | -2.670 |
| 67 | OR | 67 | -2.580 | 67 | -2.310 | 67 | -2.560 |
| 68 | OR | 69 | -2.570 | 68 | -2.280 | 68 | -2.550 |
| 69 | OR | 68 | -2.570 | 69 | -2.240 | 69 | -2.460 |
| 70 | OR | 70 | -2.550 | 70 | -2.220 | 70 | -2.400 |
| 71 | OR | 71 | -2.530 | 71 | -2.210 | 71 | -2.400 |
| 72 | OR | 72 | -2.510 | 72 | -2.200 | 72 | -2.380 |
| 73 | OR | 73 | -2.490 | 73 | -2.200 | 73 | -2.340 |
| 74 | OR | 74 | -2.480 | 74 | -2.180 | 74 | -2.260 |
| 75 | OR | 75 | -2.460 | 75 | -2.140 | 75 | -2.250 |
| 76 | OR | 76 | -2.450 | 76 | -2.130 | 76 | -2.220 |
| 77 | OR | 78 | -2.420 | 77 | -2.130 | 77 | -2.160 |
| 78 | OR | 77 | -2.420 | 78 | -2.090 | 78 | -2.130 |
| 79 | OR | 79 | -2.410 | 79 | -2.070 | 79 | -2.130 |
| 80 | OR | 80 | -2.370 | 80 | -2.060 | 80 | -2.080 |
| 81 | OR | 81 | -2.360 | 81 | -2.050 | 81 | -2.050 |
| 82 | OR | 82 | -2.330 | 82 | -2.010 | 82 | -2.030 |
| 83 | OR | 83 | -2.300 | 83 | -2.000 | 83 | -1.970 |
| 84 | OR | 84 | -2.250 | 84 | -2.000 | 85 | -1.950 |
| 85 | OR | 85 | -2.220 | 85 | -1.940 | 84 | -1.950 |
| 86 | OR | 86 | -2.160 | 86 | -1.940 | 86 | -1.920 |
| 87 | OR | 87 | -2.130 | 87 | -1.910 | 87 | -1.860 |

**Table S1.** *Cont.*

| Rank | Form | Pose | ΔG$_{bind}$ | Pose | ΔG$_{bind}$ | Pose | ΔG$_{bind}$ |
|---|---|---|---|---|---|---|---|
| 88 | OR | 88 | -2.040 | 89 | -1.880 | 88 | -1.850 |
| 89 | OR | 89 | -2.030 | 88 | -1.880 | 90 | -1.820 |
| 90 | OR | 90 | -2.010 | 90 | -1.790 | 89 | -1.820 |
| 91 | OR | 92 | -1.850 | 91 | -1.780 | 91 | -1.810 |
| 92 | OR | 91 | -1.850 | 92 | -1.770 | 92 | -1.790 |
| 93 | OR | 93 | -1.800 | 93 | -1.760 | 93 | -1.780 |
| 94 | OR | 94 | -1.540 | 94 | -1.610 | 94 | -1.660 |
| 95 | OR | 95 | -1.500 | 95 | -1.490 | 95 | -1.640 |
| 96 | OR | 96 | -1.500 | 96 | -1.450 | 96 | -1.430 |
| 97 | OR | 97 | -1.370 | 97 | -1.420 | 97 | -1.350 |
| 98 | OR | 98 | -1.340 | 98 | -1.350 | 98 | -1.100 |
| 99 | OR | 99 | -1.070 | 99 | -1.230 | 99 | -0.95 |
| 100 | OR | 100 | -0.2 | 100 | -0.7 | 100 | -0.34 |

**Figure S5.** Histograms of clustered binding conformations for open-ring (OR) form of midazolam formulated with TRIMEB. The conformational clustering was performed for three different molecular docking experiments (A–C) using a root-mean-square-deviation cut-off value of 2.0 Å.

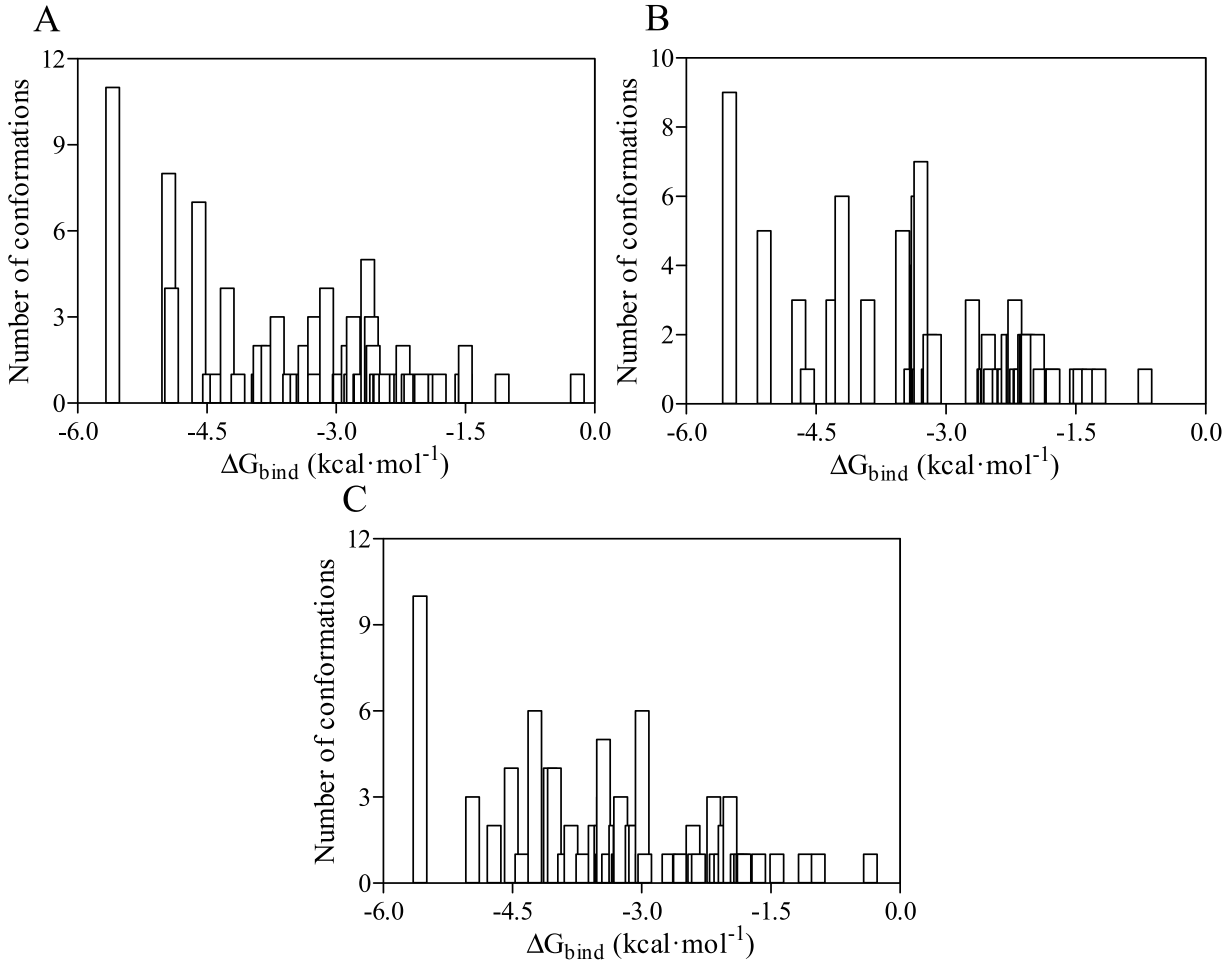

**Table S2.** Molecular docking results from three different experiments for transition state (TS) form of midazolam formulated with TRIMEB.

| Rank | Form | Pose | $\Delta G_{bind}$ | Pose | $\Delta G_{bind}$ | Pose | $\Delta G_{bind}$ |
|---|---|---|---|---|---|---|---|
| 1 | TS | 12 | -5.150 | 12 | -5.150 | 12 | -5.150 |
| 2 | TS | 13 | -5.150 | 13 | -5.150 | 13 | -5.150 |
| 3 | TS | 10 | -5.150 | 10 | -5.150 | 10 | -5.150 |
| 4 | TS | 11 | -5.150 | 11 | -5.150 | 11 | -5.150 |
| 5 | TS | 16 | -5.150 | 16 | -5.150 | 16 | -5.150 |
| 6 | TS | 17 | -5.150 | 17 | -5.150 | 17 | -5.150 |
| 7 | TS | 14 | -5.150 | 14 | -5.150 | 14 | -5.150 |
| 8 | TS | 15 | -5.150 | 15 | -5.150 | 15 | -5.150 |
| 9 | TS | 18 | -5.150 | 18 | -5.150 | 18 | -5.150 |
| 10 | TS | 19 | -5.150 | 19 | -5.150 | 19 | -5.150 |
| 11 | TS | 8 | -5.150 | 24 | -5.150 | 8 | -5.150 |
| 12 | TS | 9 | -5.150 | 25 | -5.150 | 9 | -5.150 |
| 13 | TS | 1 | -5.150 | 8 | -5.150 | 1 | -5.150 |
| 14 | TS | 2 | -5.150 | 9 | -5.150 | 2 | -5.150 |
| 15 | TS | 3 | -5.150 | 1 | -5.150 | 3 | -5.150 |
| 16 | TS | 4 | -5.150 | 2 | -5.150 | 4 | -5.150 |
| 17 | TS | 5 | -5.150 | 3 | -5.150 | 5 | -5.150 |
| 18 | TS | 6 | -5.150 | 4 | -5.150 | 6 | -5.150 |
| 19 | TS | 7 | -5.150 | 5 | -5.150 | 7 | -5.150 |
| 20 | TS | 21 | -5.150 | 6 | -5.150 | 22 | -5.150 |
| 21 | TS | 20 | -5.150 | 7 | -5.150 | 21 | -5.150 |
| 22 | TS | 24 | -5.140 | 23 | -5.150 | 20 | -5.150 |
| 23 | TS | 25 | -5.140 | 22 | -5.150 | 24 | -5.140 |
| 24 | TS | 23 | -5.140 | 21 | -5.150 | 25 | -5.140 |
| 25 | TS | 22 | -5.140 | 20 | -5.150 | 28 | -5.140 |
| 26 | TS | 26 | -5.140 | 28 | -5.140 | 30 | -5.140 |
| 27 | TS | 28 | -5.130 | 30 | -5.140 | 31 | -5.140 |
| 28 | TS | 27 | -5.130 | 31 | -5.140 | 32 | -5.140 |
| 29 | TS | 29 | -5.100 | 29 | -5.140 | 29 | -5.140 |
| 30 | TS | 30 | -4.980 | 27 | -5.140 | 23 | -5.140 |
| 31 | TS | 31 | -4.140 | 26 | -5.140 | 27 | -5.140 |
| 32 | TS | 32 | -4.120 | 32 | -5.130 | 26 | -5.140 |
| 33 | TS | 33 | -4.100 | 34 | -5.120 | 34 | -5.130 |
| 34 | TS | 38 | -4.090 | 33 | -5.120 | 35 | -5.130 |
| 35 | TS | 39 | -4.090 | 35 | -5.110 | 33 | -5.130 |
| 36 | TS | 34 | -4.090 | 36 | -5.090 | 36 | -5.110 |
| 37 | TS | 35 | -4.090 | 37 | -4.950 | 37 | -4.890 |
| 38 | TS | 36 | -4.090 | 38 | -4.910 | 38 | -4.860 |
| 39 | TS | 37 | -4.090 | 39 | -4.330 | 39 | -4.190 |
| 40 | TS | 49 | -4.080 | 40 | -4.320 | 41 | -4.100 |
| 41 | TS | 48 | -4.080 | 41 | -4.280 | 40 | -4.100 |
| 42 | TS | 45 | -4.080 | 42 | -4.230 | 45 | -4.090 |
| 43 | TS | 44 | -4.080 | 43 | -4.140 | 44 | -4.090 |

**Table S2.** *Cont.*

| Rank | Form | Pose | $\Delta G_{bind}$ | Pose | $\Delta G_{bind}$ | Pose | $\Delta G_{bind}$ |
|---|---|---|---|---|---|---|---|
| 44 | TS | 47 | -4.080 | 44 | -4.100 | 47 | -4.090 |
| 45 | TS | 46 | -4.080 | 49 | -4.090 | 46 | -4.090 |
| 46 | TS | 41 | -4.080 | 48 | -4.090 | 43 | -4.090 |
| 47 | TS | 40 | -4.080 | 45 | -4.090 | 42 | -4.090 |
| 48 | TS | 43 | -4.080 | 47 | -4.090 | 49 | -4.080 |
| 49 | TS | 42 | -4.080 | 46 | -4.090 | 48 | -4.080 |
| 50 | TS | 52 | -4.080 | 50 | -4.090 | 54 | -4.080 |
| 51 | TS | 53 | -4.080 | 56 | -4.080 | 52 | -4.080 |
| 52 | TS | 50 | -4.080 | 57 | -4.080 | 53 | -4.080 |
| 53 | TS | 51 | -4.080 | 54 | -4.080 | 50 | -4.080 |
| 54 | TS | 70 | -4.070 | 55 | -4.080 | 51 | -4.080 |
| 55 | TS | 71 | -4.070 | 52 | -4.080 | 78 | -4.070 |
| 56 | TS | 58 | -4.070 | 53 | -4.080 | 70 | -4.070 |
| 57 | TS | 59 | -4.070 | 51 | -4.080 | 71 | -4.070 |
| 58 | TS | 56 | -4.070 | 70 | -4.070 | 72 | -4.070 |
| 59 | TS | 57 | -4.070 | 71 | -4.070 | 73 | -4.070 |
| 60 | TS | 54 | -4.070 | 72 | -4.070 | 74 | -4.070 |
| 61 | TS | 55 | -4.070 | 73 | -4.070 | 75 | -4.070 |
| 62 | TS | 67 | -4.070 | 58 | -4.070 | 76 | -4.070 |
| 63 | TS | 66 | -4.070 | 59 | -4.070 | 77 | -4.070 |
| 64 | TS | 65 | -4.070 | 67 | -4.070 | 58 | -4.070 |
| 65 | TS | 64 | -4.070 | 66 | -4.070 | 59 | -4.070 |
| 66 | TS | 63 | -4.070 | 65 | -4.070 | 56 | -4.070 |
| 67 | TS | 62 | -4.070 | 64 | -4.070 | 57 | -4.070 |
| 68 | TS | 61 | -4.070 | 63 | -4.070 | 55 | -4.070 |
| 69 | TS | 60 | -4.070 | 62 | -4.070 | 67 | -4.070 |
| 70 | TS | 69 | -4.070 | 61 | -4.070 | 66 | -4.070 |
| 71 | TS | 68 | -4.070 | 60 | -4.070 | 65 | -4.070 |
| 72 | TS | 72 | -4.060 | 69 | -4.070 | 64 | -4.070 |
| 73 | TS | 73 | -4.060 | 68 | -4.070 | 63 | -4.070 |
| 74 | TS | 74 | -4.060 | 74 | -4.060 | 62 | -4.070 |
| 75 | TS | 75 | -4.040 | 75 | -4.060 | 61 | -4.070 |
| 76 | TS | 76 | -4.030 | 76 | -3.830 | 60 | -4.070 |
| 77 | TS | 77 | -4.010 | 77 | -3.720 | 69 | -4.070 |
| 78 | TS | 78 | -3.990 | 78 | -3.610 | 68 | -4.070 |
| 79 | TS | 79 | -3.980 | 79 | -3.600 | 81 | -4.060 |
| 80 | TS | 80 | -3.900 | 80 | -3.580 | 80 | -4.060 |
| 81 | TS | 81 | -3.870 | 81 | -3.420 | 79 | -4.060 |
| 82 | TS | 82 | -3.740 | 82 | -3.390 | 83 | -4.040 |
| 83 | TS | 83 | -3.680 | 83 | -3.370 | 82 | -4.040 |
| 84 | TS | 84 | -3.670 | 84 | -3.370 | 84 | -3.880 |
| 85 | TS | 85 | -3.660 | 85 | -3.260 | 85 | -3.640 |
| 86 | TS | 86 | -3.630 | 87 | -3.230 | 86 | -3.610 |
| 87 | TS | 87 | -3.440 | 86 | -3.230 | 88 | -3.590 |
| 88 | TS | 88 | -3.300 | 88 | -3.220 | 87 | -3.590 |

**Table S2.** *Cont.*

| Rank | Form | Pose | ΔG$_{bind}$ | Pose | ΔG$_{bind}$ | Pose | ΔG$_{bind}$ |
|---|---|---|---|---|---|---|---|
| 89 | TS | 89 | -3.220 | 89 | -3.210 | 89 | -3.450 |
| 90 | TS | 92 | -3.210 | 90 | -3.210 | 90 | -3.390 |
| 91 | TS | 90 | -3.210 | 92 | -3.200 | 91 | -3.360 |
| 92 | TS | 91 | -3.210 | 91 | -3.200 | 92 | -3.250 |
| 93 | TS | 93 | -3.200 | 93 | -3.190 | 93 | -3.210 |
| 94 | TS | 94 | -3.190 | 94 | -3.190 | 94 | -3.200 |
| 95 | TS | 95 | -3.180 | 95 | -3.190 | 95 | -3.190 |
| 96 | TS | 96 | -3.170 | 96 | -3.090 | 96 | -3.180 |
| 97 | TS | 97 | -3.170 | 97 | -3.080 | 97 | -3.170 |
| 98 | TS | 98 | -3.160 | 98 | -3.000 | 98 | -3.080 |
| 99 | TS | 99 | -3.120 | 99 | -3.000 | 99 | -3.000 |
| 100 | TS | 100 | -3.040 | 100 | -2.990 | 100 | -2.460 |

**Figure S6.** Histograms of clustered binding conformations for transition state (TS) form of midazolam formulated with TRIMEB. The conformational clustering was performed for three different molecular docking experiments (A–C) using a root-mean-square-deviation cut-off value of 2.0 Å.

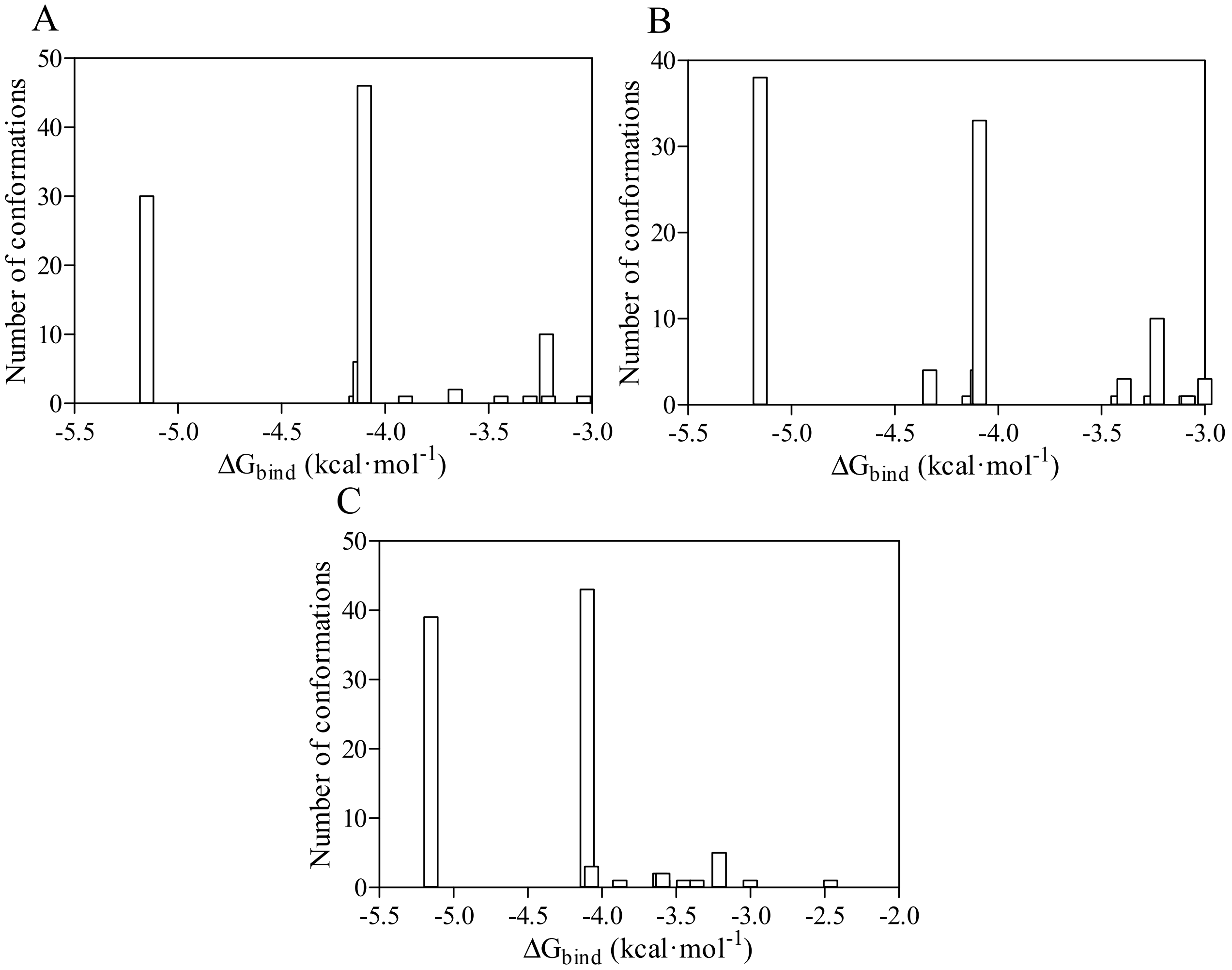

**Table S3.** Molecular docking results from three different experiments for closed-ring (CR) form of midazolam formulated with TRIMEB.

| Rank | Form | Pose | $\Delta G_{bind}$ | Pose | $\Delta G_{bind}$ | Pose | $\Delta G_{bind}$ |
|---|---|---|---|---|---|---|---|
| 1 | CR | 3 | -5.150 | 3 | -4.410 | 12 | -4.410 |
| 2 | CR | 2 | -5.150 | 2 | -4.410 | 11 | -4.410 |
| 3 | CR | 1 | -5.150 | 1 | -4.410 | 10 | -4.410 |
| 4 | CR | 13 | -5.140 | 7 | -4.410 | 3 | -4.410 |
| 5 | CR | 12 | -5.140 | 6 | -4.410 | 2 | -4.410 |
| 6 | CR | 11 | -5.140 | 5 | -4.410 | 1 | -4.410 |
| 7 | CR | 10 | -5.140 | 4 | -4.410 | 7 | -4.410 |
| 8 | CR | 7 | -5.140 | 9 | -4.410 | 6 | -4.410 |
| 9 | CR | 6 | -5.140 | 8 | -4.410 | 5 | -4.410 |
| 10 | CR | 5 | -5.140 | 17 | -4.400 | 4 | -4.410 |
| 11 | CR | 4 | -5.140 | 16 | -4.400 | 9 | -4.410 |
| 12 | CR | 9 | -5.140 | 15 | -4.400 | 8 | -4.410 |
| 13 | CR | 8 | -5.140 | 14 | -4.400 | 17 | -4.400 |
| 14 | CR | 17 | -5.130 | 13 | -4.400 | 16 | -4.400 |
| 15 | CR | 16 | -5.130 | 12 | -4.400 | 15 | -4.400 |
| 16 | CR | 15 | -5.130 | 11 | -4.400 | 14 | -4.400 |
| 17 | CR | 14 | -5.130 | 10 | -4.400 | 13 | -4.400 |
| 18 | CR | 19 | -5.130 | 19 | -4.400 | 19 | -4.400 |
| 19 | CR | 18 | -5.130 | 18 | -4.400 | 18 | -4.400 |
| 20 | CR | 20 | -5.130 | 20 | -4.400 | 20 | -4.400 |
| 21 | CR | 24 | -5.120 | 24 | -4.390 | 21 | -4.400 |
| 22 | CR | 25 | -5.120 | 25 | -4.390 | 24 | -4.390 |
| 23 | CR | 22 | -5.120 | 22 | -4.390 | 25 | -4.390 |
| 24 | CR | 23 | -5.120 | 23 | -4.390 | 28 | -4.390 |
| 25 | CR | 21 | -5.120 | 21 | -4.390 | 22 | -4.390 |
| 26 | CR | 26 | -5.120 | 26 | -4.380 | 23 | -4.390 |
| 27 | CR | 28 | -5.110 | 27 | -4.380 | 26 | -4.390 |
| 28 | CR | 27 | -5.110 | 28 | -4.370 | 27 | -4.390 |
| 29 | CR | 29 | -5.110 | 30 | -4.370 | 31 | -4.380 |
| 30 | CR | 31 | -5.100 | 29 | -4.370 | 30 | -4.380 |
| 31 | CR | 30 | -5.100 | 35 | -4.360 | 32 | -4.380 |
| 32 | CR | 32 | -5.070 | 31 | -4.360 | 29 | -4.380 |
| 33 | CR | 33 | -5.050 | 33 | -4.360 | 33 | -4.370 |
| 34 | CR | 34 | -5.040 | 32 | -4.360 | 35 | -4.360 |
| 35 | CR | 36 | -4.990 | 34 | -4.360 | 34 | -4.360 |
| 36 | CR | 35 | -4.990 | 36 | -4.340 | 36 | -4.350 |
| 37 | CR | 37 | -4.990 | 37 | -4.340 | 37 | -4.350 |
| 38 | CR | 38 | -4.990 | 38 | -4.340 | 38 | -4.350 |
| 39 | CR | 39 | -4.950 | 39 | -4.330 | 39 | -4.350 |
| 40 | CR | 53 | -4.930 | 40 | -4.330 | 40 | -4.350 |
| 41 | CR | 52 | -4.930 | 41 | -4.330 | 44 | -4.340 |
| 42 | CR | 51 | -4.930 | 42 | -4.330 | 45 | -4.340 |
| 43 | CR | 50 | -4.930 | 43 | -4.330 | 46 | -4.340 |

**Table S3.** *Cont.*

| Rank | Form | Pose | ΔG$_{bind}$ | Pose | ΔG$_{bind}$ | Pose | ΔG$_{bind}$ |
|---|---|---|---|---|---|---|---|
| 44 | CR | 55 | -4.930 | 44 | -4.320 | 41 | -4.340 |
| 45 | CR | 54 | -4.930 | 45 | -4.320 | 42 | -4.340 |
| 46 | CR | 44 | -4.930 | 46 | -4.320 | 43 | -4.340 |
| 47 | CR | 45 | -4.930 | 47 | -4.320 | 52 | -4.330 |
| 48 | CR | 46 | -4.930 | 48 | -4.320 | 51 | -4.330 |
| 49 | CR | 47 | -4.930 | 51 | -4.310 | 50 | -4.330 |
| 50 | CR | 40 | -4.930 | 50 | -4.310 | 47 | -4.330 |
| 51 | CR | 41 | -4.930 | 49 | -4.310 | 48 | -4.330 |
| 52 | CR | 42 | -4.930 | 53 | -4.300 | 49 | -4.330 |
| 53 | CR | 43 | -4.930 | 52 | -4.300 | 53 | -4.320 |
| 54 | CR | 48 | -4.930 | 57 | -4.300 | 56 | -4.320 |
| 55 | CR | 49 | -4.930 | 56 | -4.300 | 55 | -4.320 |
| 56 | CR | 65 | -4.920 | 55 | -4.300 | 54 | -4.320 |
| 57 | CR | 57 | -4.920 | 54 | -4.300 | 57 | -4.310 |
| 58 | CR | 56 | -4.920 | 58 | -4.300 | 59 | -4.310 |
| 59 | CR | 59 | -4.920 | 65 | -4.290 | 58 | -4.310 |
| 60 | CR | 58 | -4.920 | 59 | -4.290 | 60 | -4.310 |
| 61 | CR | 92 | -4.920 | 71 | -4.290 | 65 | -4.300 |
| 62 | CR | 91 | -4.920 | 70 | -4.290 | 68 | -4.300 |
| 63 | CR | 90 | -4.920 | 68 | -4.290 | 66 | -4.300 |
| 64 | CR | 78 | -4.920 | 69 | -4.290 | 67 | -4.300 |
| 65 | CR | 79 | -4.920 | 66 | -4.290 | 64 | -4.300 |
| 66 | CR | 74 | -4.920 | 67 | -4.290 | 62 | -4.300 |
| 67 | CR | 77 | -4.920 | 64 | -4.290 | 63 | -4.300 |
| 68 | CR | 76 | -4.920 | 62 | -4.290 | 61 | -4.300 |
| 69 | CR | 71 | -4.920 | 63 | -4.290 | 74 | -4.290 |
| 70 | CR | 70 | -4.920 | 60 | -4.290 | 76 | -4.290 |
| 71 | CR | 73 | -4.920 | 61 | -4.290 | 71 | -4.290 |
| 72 | CR | 72 | -4.920 | 78 | -4.280 | 70 | -4.290 |
| 73 | CR | 80 | -4.920 | 74 | -4.280 | 73 | -4.290 |
| 74 | CR | 81 | -4.920 | 77 | -4.280 | 72 | -4.290 |
| 75 | CR | 82 | -4.920 | 76 | -4.280 | 69 | -4.290 |
| 76 | CR | 83 | -4.920 | 73 | -4.280 | 75 | -4.290 |
| 77 | CR | 84 | -4.920 | 72 | -4.280 | 78 | -4.280 |
| 78 | CR | 85 | -4.920 | 75 | -4.280 | 79 | -4.280 |
| 79 | CR | 86 | -4.920 | 79 | -4.270 | 77 | -4.280 |
| 80 | CR | 87 | -4.920 | 80 | -4.270 | 80 | -4.280 |
| 81 | CR | 88 | -4.920 | 81 | -4.250 | 81 | -4.280 |
| 82 | CR | 89 | -4.920 | 82 | -4.240 | 82 | -4.280 |
| 83 | CR | 68 | -4.920 | 83 | -4.230 | 83 | -4.280 |
| 84 | CR | 69 | -4.920 | 84 | -4.230 | 84 | -4.280 |
| 85 | CR | 66 | -4.920 | 85 | -4.220 | 85 | -4.280 |
| 86 | CR | 67 | -4.920 | 86 | -4.220 | 86 | -4.280 |
| 87 | CR | 64 | -4.920 | 87 | -4.200 | 87 | -4.270 |
| 88 | CR | 62 | -4.920 | 88 | -3.940 | 92 | -4.260 |

**Table S3.** *Cont.*

| Rank | Form | Pose | $\Delta G_{bind}$ | Pose | $\Delta G_{bind}$ | Pose | $\Delta G_{bind}$ |
|---|---|---|---|---|---|---|---|
| 89 | CR | 63 | -4.920 | 89 | -3.630 | 91 | -4.260 |
| 90 | CR | 60 | -4.920 | 90 | -3.620 | 90 | -4.260 |
| 91 | CR | 61 | -4.920 | 91 | -3.590 | 88 | -4.260 |
| 92 | CR | 75 | -4.920 | 92 | -3.570 | 89 | -4.260 |
| 93 | CR | 96 | -4.910 | 93 | -3.460 | 93 | -4.250 |
| 94 | CR | 93 | -4.910 | 94 | -3.460 | 94 | -4.250 |
| 95 | CR | 95 | -4.910 | 95 | -3.450 | 95 | -4.240 |
| 96 | CR | 94 | -4.910 | 97 | -3.440 | 96 | -4.090 |
| 97 | CR | 97 | -4.840 | 96 | -3.440 | 97 | -3.620 |
| 98 | CR | 98 | -4.770 | 98 | -3.430 | 98 | -3.450 |
| 99 | CR | 99 | -4.760 | 99 | -3.390 | 99 | -3.310 |
| 100 | CR | 100 | -4.720 | 100 | -3.340 | 100 | -2.980 |

**Figure S7.** Histograms of clustered binding conformations for closed-ring (CR) form of midazolam formulated with TRIMEB. The conformational clustering was performed for three different molecular docking experiments (A–C) using a root-mean-square-deviation cut-off value of 2.0 Å.

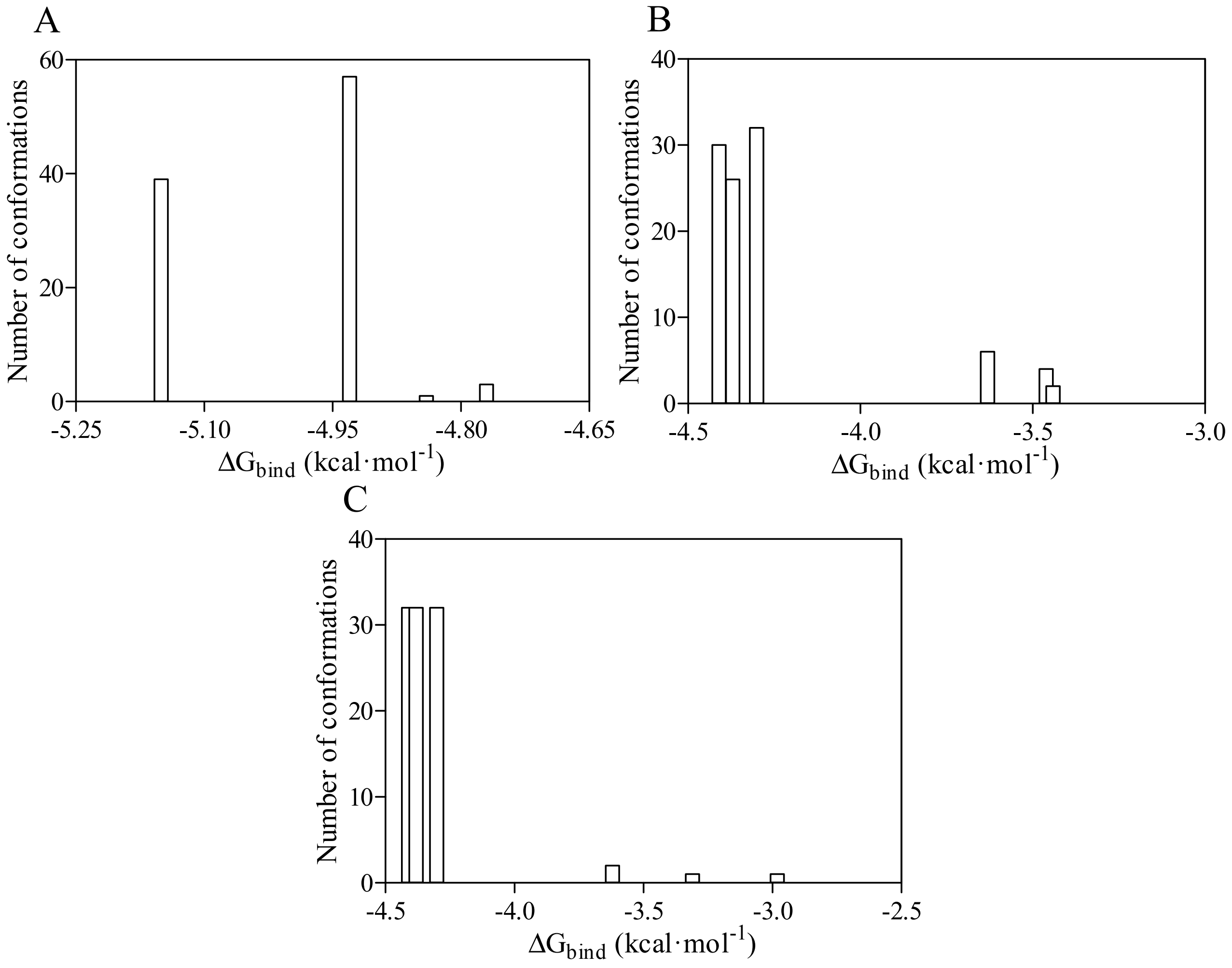

Supplement: Supplementary File 1 [file molecules-19-16861-s001.zip › Supplementary Material 3.pdf]
